# 

**Published:** 1899-03

**Authors:** 


					﻿Southern Medical Record
A Monthly Journal of Medicine and Surgery.
Vol. XXDČ	ATLANTA, GA., MARCH, 1899.	No. 3/
BERNARD WOLFF, M. D., Editor.
Associate Editors :
A. W. GRIGGS, M. D.	J. McFADDEN GASTON, M. D.
J. McFADDEN GASTON, Jr., M. D.	WILLIS F. WESTMORELAND, M. D
MICHAEL HOKE, M.D., Business Manager.
CHAS. ROOME PARMELE CO., 36 Platt Street, new YORK.
				

